# Predisposing factors for a second fragile hip fracture in a population of 1130 patients with hip fractures, treated at Oulu University Hospital in 2013–2016: a retrospective study

**DOI:** 10.1007/s00402-022-04406-4

**Published:** 2022-03-15

**Authors:** Nelli Helynen, Lotta Rantanen, Petri Lehenkari, Maarit Valkealahti

**Affiliations:** 1 Division of Orthopedic and Trauma Surgery and Medical Research Center, Department of Surgery, Faculty of Medicine, University of Oulu, Oulu, Finland; 2 Department of Anatomy and Cell Biology, Faculty of Medicine, University of Oulu, Oulu, Finland; 3 Division of Operative Care, Oulu University Hospital and Medical Research Center Oulu, University of Oulu, Oulu, Finland

**Keywords:** Osteoporosis, Fragile hip fracture, Bone turnover markers, High bone turn over, Vitamin D, BMD, Screening of osteoporosis

## Abstract

**Objective:**

The life-time risk of a second fragile hip fracture is 8.4%, but the risk factors that predispose to a second hip fracture remain unresolved. This study aimed to define risk factors that predisposed patients to a second hip fracture.

**Methods:**

For this retrospective study, we retrieved clinical data on 1130 patients with fragile hip fractures (67.2% female, mean age: 79.3 years) that underwent surgery at the Oulu University Hospital in 2013–2016. These data included the fracture risk assessment score (measured with the FRAX tool), the bone-mass *T*-score, laboratory values, ambulatory capacity, and the time of death.

**Results:**

In this population, 12.4% of patients sustained a second hip fracture. The predisposing factors for a second hip fracture were: female (*p* = 0.016), a high FRAX score (*p* = 0.020), and low physical capacity (*p* < 0.001). The vitamin D level recommended for treating osteoporosis (i.e., vitamin D > 75 nmol/l) was observed in only 24% of patients, and 42% of patients had ionized calcium levels below the reference range. According to the level of the cross-linked carboxy-terminal telopeptide of type I collagen (ICTP), 37% of patients did not have high bone turnover. We found a positive correlation between age and ICTP (*p* = 0.001). The risk of death was higher after the second hip fracture (*p* = 0.005), but we found no difference in age between patients with first and second hip fractures (*p* = 0.11).

**Conclusion:**

After a hip fracture, a second hip fracture is a well-known risk. Nevertheless, we found that only one-third of patients with a second hip fracture had used anti-osteoporosis medication at any time previously. These findings suggested that second hip fractures were most likely to occur in patients with osteopenic *T*-score values, in women more often than men, and in patients with high FRAX scores and low ambulatory capacity.

## Introduction

Osteoporosis affects hundreds of millions of people worldwide, particularly postmenopausal women. The impact of osteoporosis is expected to rise in future, due to the increasing proportion of older individuals in the population. The main clinical consequence of osteoporosis is an increase in the risk of fragile fractures [1, 2]. It is estimated that 18% of women and 6% of men worldwide will sustain hip fractures during their life span. The number of fractures is expected to increase from 1.26 million, in 1990, to 4.5 million by the year 2050 [3].

Osteoporosis is characterized by a decline in bone strength, a disruption in bone architecture, and a reduction in bone mass [1, 4]. Bone quality is evaluated in terms of mineralization, microdamage accumulation, microarchitecture, and the bone turnover rate. Currently, the only measurable factor included in the definition of osteoporosis is bone mass. Bone mass is mainly expressed as the bone mineral density (BMD), which is measured with dual X-ray absorptiometry (DXA) [2, 5]. According to the World Health Organization (WHO), osteoporosis is defined as a BMD T-score that is 2.5 standard deviations (SD) below the BMD of a young female adult [6–8]. In addition to the BMD, the fracture risk assessment (FRAX), estimated with an FRAX-risk calculator, is typically used to determine treatment strategies [9].

Despite the worldwide use of FRAX and DXA, these modalities have several limitations. First, they do not capture the risk of fragile fractures or consider medications that influence the risk of fractures [2, 9, 10]. Second, they completely ignore the effects of bone turnover markers, which are biochemical byproducts of bone resorption and bone formation [9–11]. Several studies have provided evidence in support of the clinical application of bone turnover markers for the treatment of osteoporosis and for predictions of fracture risk. However, it has not been conclusively determined how bone turnover markers can be utilized in practice [2, 9, 12, 13]. Monitoring these markers may provide clinically useful information for managing patients with fragile fractures [13–15].

High bone turnover is associated with an increased risk of fractures [16, 17]. The levels of two bone turnover markers, the N-terminal propeptide of type I procollagen (PINP) and the cross-linked carboxy-terminal telopeptide of type I collagen (ICTP), increase after a fracture [14, 18, 19]. However, further research is required to determine baseline levels of bone turnover markers among postmenopausal women with osteoporosis and in a healthy population.

In Finland, 72% of the general population have vitamin D levels above 50 nmol/l [20]. Low levels of vitamin D are common in older people, and vitamin D deficiencies may lead to bone loss, increased bone resorption, fractures, and falls. However, studies that evaluated the effects of vitamin D on BMD have yielded conflicting results [21]. Thus, it remains unclear whether low levels of D-25-OH concentrations predispose individuals to fragile fractures [22]. According to different studies, either low or high D-25-OH concentrations can increase bone resorption [23]. The protective effects of vitamin D supplementation on bone health, the risk of falls, and mortality are often observed in individuals with low D-25-OH concentrations [24, 25].

Conflicting results have also been reported on the effects of anti-resorptive drugs. Some studies suggest that anti-resorptive medications can effectively prevent fractures in patients at high risk. In contrast, according to other studies, anti-resorptive medications had no effect on the risk of a second hip fracture [26, 27].

Overall, according to a systematic review, the life-time risk of a second hip fracture is 8.4% in patients that experienced one hip fracture [28]. The bone turnover marker, alkaline phosphatase (ALP), was positively associated with the incidence of fragile hip fractures, and low ALP levels were associated with reduced mortality [29, 30]. Mortality was also found to be associated with elevated calcium levels, but not with parathyroid hormone (PTH) levels [31]. Bone turnover markers, ALP, and tartrate-resistant acid phosphatase-5b were negatively correlated with BMD. In addition, BMD was positively correlated with the body mass index (BMI). According to some studies, obesity reduced the risk of hip fractures and the risk of falling. Physical exercise also had positive effects on BMD [32–36].

Monitoring different markers may provide clinically useful information for managing patients with fragile fractures. The present study aimed to investigate the relationship between the incidence of fragile hip fractures and different parameters, including bone turnover markers. We determined the baseline levels of different parameters at the time of a fracture, and then, we investigated the effects of these parameters on the incidence of a second hip fracture. We hypothesized that the D-25-OH and Ca-ion levels would be lower in patients that had sustained a fragile hip fracture, compared to the general population, and that elevated bone resorption would not be observed in all patients with fragile hip fractures.

## Patients and methods

For this retrospective study, we retrieved data on all patients that were admitted to the orthopedic ward of Oulu University Hospital with a fragile hip fracture and were treated with osteosynthesis or arthroplasty, between January 2013 and December 2016. In the fall of 2019, we retrospectively identified 1130 patients. The Ethics Committee of Oulu University Hospital granted a research permit (108/2016).

We collected data on the following variables: the patient’s diagnosis, sex, age, and BMI; the time between the last hip fracture and death; the number of low-energy fractures; the time between first and second hip fractures; the FRAX score; the risk of a major fracture and the risk of a hip fracture; and the DXA measurement, including the BMD and *T*-score. Some patients had *T*-scores measured in both hips. For the present study, we analyzed the lower *T*-score between the two hip measurements (i.e., the lowest hip *T*-score). We also collected data from laboratory tests, including: the levels of bone turnover markers, PINP and ICTP; and the levels of PTH, D-25-OH, ionized calcium (aB-Ca-ion), adjusted ionized calcium at pH 7.4 (aB-Ca-I7.4), and alkaline phosphatase (P-ALP). We collected the values of PTH and D25-OH measured at the time of fracture (baseline values) and during follow-up (post-operative values) at 6, 12, and 24 months. Bone turn over values were collected only from a few patients (PINP *n* = 39 and ICTP *n* = 35), because they were advised to measure within 24 h from the fracture event and there have been delays in admission to the hospital, and simple human oblivitions.

Patients were divided into the following groups, based on their ambulatory capacity at the time of the incident: (1) required assistive devices/ambulatory aids or lived in an institutional care facility, (2) could walk, but only up to one kilometer, and (3) fully ambulatory. The records indicated whether the patient had used osteoporosis medications at any time or at the time of the fracture. The records also included the use of bisphosphonates, denosumab, strontium ranelate, teriparatide, vitamin D, calcium, or combined supplementation. In the surgery clinic, a nurse specialized in osteoporosis screened patients with fragile hip fractures for osteoporosis. However, due to the lack of resources, patients with fragile hip fractures could not be screened systematically. Furthermore, the clinic tended to omit DXA measurements for older patients with many comorbidities.

All statistical analyses were performed with IBM SPSS Statistics Data Editor 26.0 software. Distributions of continuous variables were tested with the Kolmogorov–Smirnov test and histogram analyses. For correlation analyses, we used Spearman’s correlate for skewed data distributions and Pearson’s correlation for normal data distributions. The correlation coefficients were interpreted as follows: values ≤ 0.35 represented weak correlations, values of 0.36–0.67 represented moderate correlations, and values of 0.68–1.0 represented strong correlations. *p *Values < 0.05 were deemed statistically significant. Between-group comparisons for continuous variables were analyzed with the Kruskal–Wallis test, two-sample *t* test, or ANOVA. Categorical variables were compared with cross-tabulation tables, and the Pearson chi-square test was used to evaluate the two-tailed statistical significance of cross-tabulations.

## Results

All the patients included in this study (67.2% female, 32.8% male; mean age, 79.3 years) had at least one low-energy hip fracture. We observed the following types of fractures, based on the International Classification of Disease, 10th revision (ICD10) codes: neck of femur (ICD10: S72.0, 57.7%), pertrochanteric (ICD10: S72.1, 35.1%), and subtrochanteric (ICD10: S72.2, 7.2%).

The mean measured values were: BMI = 24.3 kg/m^2^, vitamin D = 59.8 nmol/l, and ionized calcium = 1.2 mmol/l. Among the 1077 patients with fragile hip fractures, 51.9% had lived in an institutional care facility or required ambulatory aids (Table 1).

**Table 1:** Initial patient characteristics

Characteristic	*N*_total_ = 1130	%	Mean	SD
Age (years)	1130​		79.3​	11.0​
Age stratified by sex (years)				
Female	759	67.2	81.4​	9.4​
Male	371​	32.8	74.9	12.6​
Weight​ (kg)	1012​		66.0​	13.8​
Height​ (cm)	993​		164.8​	9.2​
BMI​ (kg/m^2^)	993​		24.3​	4.3​
D-25-OH ​(nmol/l)	349​		59.8​	20.3​
aB-Ca-Ion (mmol/l) ​	511		1.2​	0.08​
aB-Ca-l7.4​ (mmol/l)	510		1.2​	0.08​
ALP ​(U/l)	459		85.3​	41.9​
Degree of ambulation before the fracture​	1077		1.7​	0.8​
1​	586	51.9​		
2​	228	20.2​		
3​	263	23.3​		
Diagnosis​^a^	1130​			
0 (S72.0)​	652​	57.7​		
1 (S72.1)​	397​	35.1​		
2 (S72.2)​	81​	7.2​		
Anti-resorptive drug use	323​			
Fragile fractures​	1122​		1.6​	1.0​
Time between 1st and 2nd hip fractures​ (months)	128​		48.2​	48.7​

*BMI* body mass index, *aB-Ca-Ion* ionized calcium, *aB-Ca-I7.4* adjusted ionized calcium at pH 7.4, *ALP* alkaline phosphatase

^a^International Classification of Disease, 10th revision codes for hip fracture diagnoses: S72.0-Fracture of head and neck of femur; S72.1-Pertrochanteric fracture; S72.2-Subtrochanteric fracture of femur

The mean *T*-scores were − 2.49 (SD 0.88), − 2.43 (SD 1.35), and − 1.47 (SD 1.81), for fractures in the neck of the femur (*n* = 64), pertrochanteric fractures (*n* = 33), and subtrochanteric hip fractures (*n* = 12), respectively (*p* = 0.022).

### Factors related to the second hip fracture

Among the 1130 patients with fragile hip fractures, 128 (11.3%) had sustained a second hip fracture. The mean time interval between the two fractures was 48.2 months (SD 48.7). Among those with only one hip fracture, 661 were female (66.0%), and 341 were male (34.0%). Among the 128 patients with a second hip fracture, the majority (76.6%) was female (*n* = 98; *p* = 0.016). The lowest hip *T*-score was significantly lower for the first hip fracture compared to the second hip fracture (*p* = 0.016). The risk of a hip fracture, based on the FRAX scores, was significantly lower among patients with a first hip fracture (mean FRAX = 10.2), compared to patients with a second hip fracture (mean FRAX = 19.4; *p* = 0.020).

The distribution of fracture sites was different between the first and second hip fractures. The proportion of subtrochanteric fractures was greater in the group of second hip fractures (13.3%) than in the group of first hip fractures (6.4%; *p* = 0.014). In both groups, the majority of patients had neck of femur fractures (first fracture: 58.5%; second fracture: 51.6%). In both groups, 35% of patients had pertrochanteric fractures.

The use of anti-resorptive drugs differed in the first and second hip fracture groups. Anti-resorptive drugs were used at some time by 28.8% (*n* = 289) of patients with first hip fractures and by 32.0% (*n* = 41) of patients with second hip fractures. However, at the time of the first hip fracture, only 4.7% of patients used anti-resorptive medications. Ambulatory capacity before a first hip fracture was significantly lower among patients who later sustained a second hip fracture (*p* < 0.001). The majority (68%) of patients with second hip fractures lived in a nursing home or used a walking aid (Table 2).

**Table 2:** Risk assessment for a second hip fracture

Variable	First hip fracture (*N* = 1002)			Second hip fracture (*N* = 128)			*p* Value
	*N* (%)	Mean	SD	*N* (%)	Mean	SD	
Sex, F/M	661/341			98/30			0.016
Age, years	1002	79.1	11.0	128	80.8	11.0	0.11
BMI, kg/m^2^	884	24.4	4.4	109	23.7	4.2	0.13
Lowest hip *T*-score^a^	105	– 2.4	1.1	4	– 1.0	1.8	0.016
BMD (L1-L4)	133	1.1	0.2	15	1.0	0.2	0.26
FRAX	44	10.2	9.2	7	19.4	11.4	0.020
Diagnosis^b^							0.014
S72.0	586 (58.5)			66 (51.6)			
S72.1	352 (35.1)			45 (35.2)			
S72.2	64 (6.4)			17 (13.3)			
Physical exercise^c^							< 0.001
1	499 (49.8)			87 (68.0)			
2	207 (20.7)			21 (16.4)			
3	248 (24.8)			15 (11.7)			
Vitamin D use	259 (25.8)			46 (35.9)			0.015
Anti-resorptive drug	289 (28.8)			41 (32.0)			0.46
Exitus	503 (50.2)			81 (63.3)			0.005

*BMI* body mass index, *BMD (L1-L4)* bone mineral density of lumbar vertebrae 1 through 4; *FRAX* fracture risk assessment tool

^a^Lowest hip *T*-score: Some patients had *T*-scores measured in both hips. For the present study, we analyzed the lower *T*-score between the two hip measurements (i.e., the lowest hip *T*-score)

^b^International Classification of Disease, 10th revision codes for hip fracture diagnoses: S72.0-Fracture of head and neck of femur; S72.1-Pertrochanteric fracture; S72.2-Subtrochanteric fracture of femur

^c^Physical exercise was defined as: (1) required assistive devices/ambulatory aids or lived in an institutional care facility, (2) could walk, but only up to 1 km, and (3) fully ambulatory

### Positive effects of vitamin D were not detected

Patients were divided into two groups, based on their baseline levels of vitamin D: a low vitamin D level was defined as ≤ 40 nmol/l; a high vitamin D level was defined as > 40 nmol/l. In general, patients with low vitamin D levels had lower ICTP and PINP values, but the differences between groups were not statistically significant. The number of low-energy fractures was higher in the high vitamin D group than in the low vitamin D group (*p* = 0.003). The mean *T*-score of patients with low vitamin D (− 2.0) was higher than that of patients with high vitamin D (− 2.5). The mean of BMD of the lumbar vertebrae (L1-L4) was approximately the same in both groups. In the low vitamin D group, 38.3% (*n* = 18) used ambulatory aids, 29.8% could walk up to 1 km (*n* = 14), and 31.9% were fully ambulatory (*n* = 15). In the high vitamin D group, these proportions were 57.8% (*n* = 166), 22.6% (*n* = 65), and 19.5% (*n* = 56), respectively (*p* = 0.037).

The mean baseline vitamin D level at the time of the hip fracture was 59.8 nmol/l (*n* = 349). The mean vitamin D levels at 6, 12, and 24 months after the fracture were 75.2 nmol/l (*n* = 97), 76.1 nmol/l (*n* = 91), and 73.3 nmol/l (*n* = 99), respectively.

The PTH levels tended to rise post-operatively. The mean baseline PTH level was 83.9 ng/l (*n* = 204). The mean PTH values at 6, 12, and 24 months after the fracture were 86.4 ng/l (*n* = 39), 92.1 ng/l (*n* = 42), and 106.8 ng/l (*n* = 39), respectively.

### Vitamin D, ionized calcium, and bone turnover

**Fig. 1:**
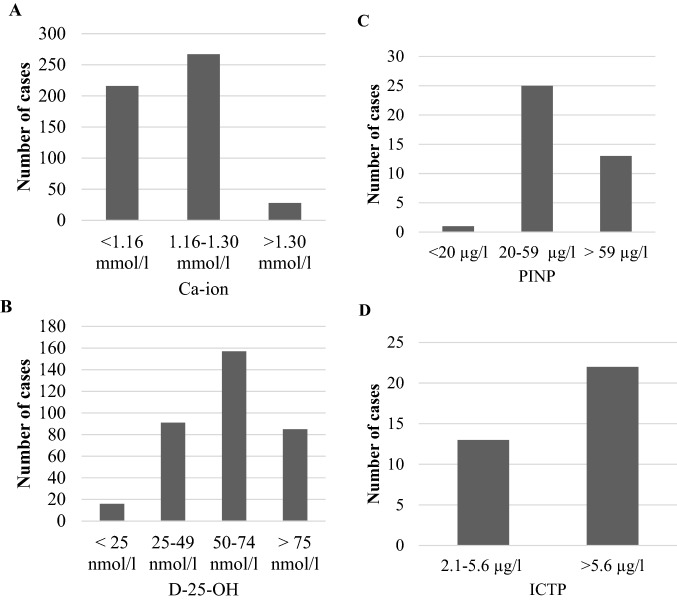
Initial characteristics of patients with fragile hip fractures. Ionized calcium (*n* = 511), Vitamin D (*n* = 349), PINP (*n *= 39), and ICTP (*n* = 35) distributions are normalized to set laboratory values. **A** 42% of patients had ionized calcium levels below the reference value (< 1.16 nmol/l). **B** 24% (*n* = 85) of patients had vitamin D levels above the level at which osteoporosis treatment is recommended (> 75 nmol/l). **C** One-third of the patients had PINP levels above 59 μg/l, and **D** 37% of patients had a normal bone turnover rate, according to the ICTP value

Ionized calcium was measured in 511 patients. Over half of these patients (*n* = 267) had values within the reference range (1.16–1.30 mmol/l). High ionized calcium levels (> 1.30 mmol/l) were detected in 28 patients. Nearly half the patients (*n* = 216) had calcium levels below the reference range (< 1.16 mmol/l; Fig. 1A).

Vitamin D (D-25-OH) levels were measured in 349 patients. Most of these patients had vitamin D levels of 50–74 nmol/l. Some patients had low levels, 25–49 nmol/l (*n* = 91). Sixteen patients had a vitamin D deficiency, defined as vitamin D levels below 25 nmol/l. Others (*n* = 85) had high levels, above the recommended threshold for treating osteoporosis (> 75 nmol/l) (Fig. 1B).

PINP was measured in only 39 patients. Normal bone formation was indicated when the PINP value fell within the reference values (20–59 μg/l). Thus, normal bone formation was observed in 25 patients. One-third of the patients had PINP levels over 59 μg/l, and one patient had a PINP level below 20 μg/l (Fig. 1C). ALP was measured in 459 patients. The highest values were observed in patients that used ambulatory aids or lived in an institutional care facility (*p* = 0.027, Kruskal–Wallis).

ICTP was measured in only 35 patients. Over one-third of these patients (37%) had normal bone turnover rates, according to the ICTP value*.* High ICTP levels (> 5.6 μg/l) were detected in 22 patients, and normal levels (2.1–5.6 μg/l) were observed in 13 patients (Fig. 1D).

### Lack of correlations with hip *T*-score

**Fig. 2:**
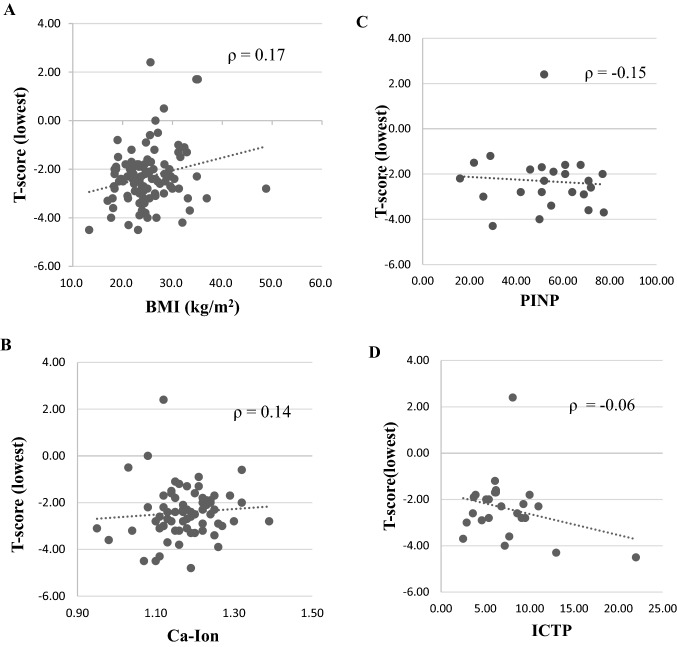
Correlations between the hip *T*-score and BMI, bone turnover markers, and calcium values. The hip *T*-score was not significantly correlated with **A** the BMI (Spearman correlation = 0.17; *p* = 0.080), **B** ionized calcium (Spearman correlation = 0.14; *p* = 0.24), **C** PINP (Spearman correlation =  − 0.15; *p* = 0.49), or **D** ICTP (Spearman correlation =  − 0.06; *p* = 0.79)

The lowest hip *T*-score was not correlated with the BMI (*p* = 0.080; Spearman correlation = 0.17; Fig. 2A), with ionized calcium (*p* = 0.24; Spearman correlation = 0.14; Fig. 2B), or with the PINP (*p* = 0.49; Spearman correlation =  − 0.15; Fig. 2C). Furthermore, there was no correlation (*ρ* =  − 0.06) between the lowest hip *T*-score and the ICTP level (*p* = 0.79; Fig. 2D).

### Relationships between age and bone turnover markers, vitamin D, and PTH

**Fig. 3:**
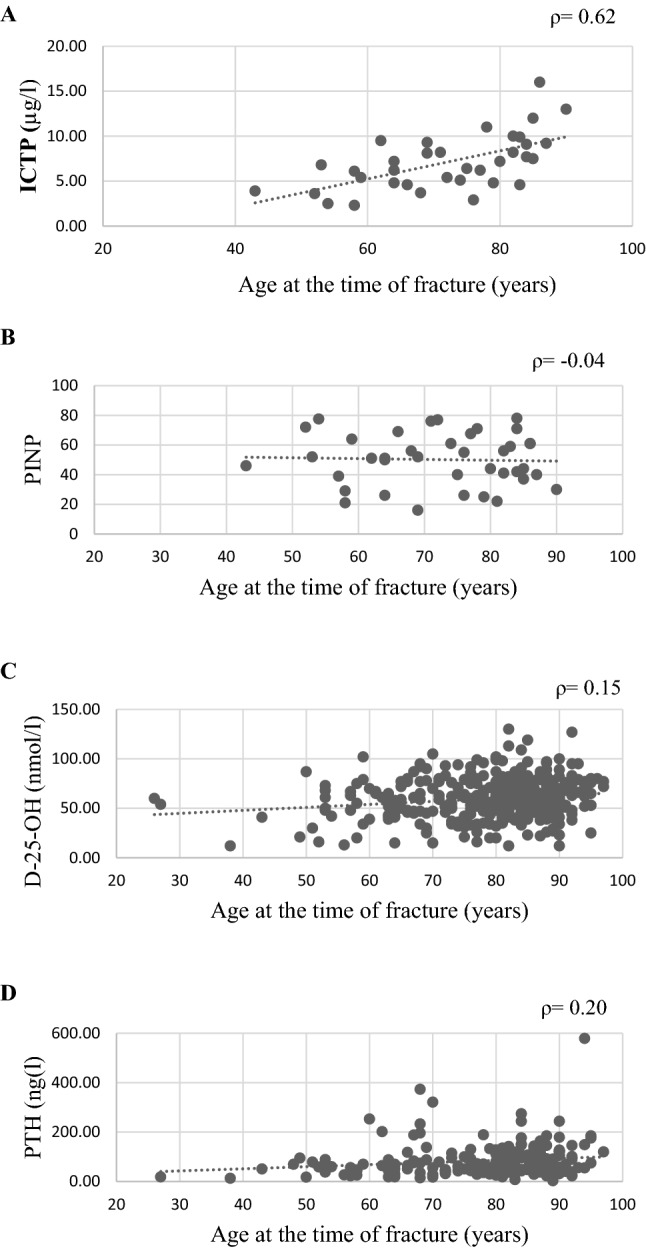
Correlations between age and bone turnover markers (PINP *n* = 39, ICTP *n* = 35), vitamin D (*n* = 349), and PTH (*n* = 204). **A** Age and ICTP were significantly correlated (Spearman correlation = 0.62; *p* < 0.001). The youngest patient with a fragile hip fracture that underwent an ICTP measurement was 43 years old, and the oldest was 90 years old. **B** age was not significantly correlated to PINP (Spearman correlation =  −  0.04, p = 0.823), **C** weak correlations were observed between age and vitamin D levels (Spearman correlation = 0.15, *p* = 0.004) and **D** between age and PTH levels (*ρ* = 0.20, *p* = 0.004)

Among patients with fragile hip fractures that underwent ICTP measurements, the youngest was 43 years old and the oldest was 90 years old. A moderate positive correlation (*ρ* = 0.62) was observed between age and ICTP levels (*p* < 0.001; Fig. 3A). In contrast, age was not significantly correlated to PINP (*ρ* =  − 0.04, *p* = 0.823; Fig. 3B). Weak correlations were observed between age and vitamin D levels (*ρ* = 0.15, *p* = 0.004; Fig. 3C) and between age and PTH levels (*ρ* = 0.20, *p* = 0.004; Fig. 3D).

### Height and weight effects on bone strength

Height showed a weak positive correlation with BMD L1–L4 (*ρ* = 0.21, *p* < 0.05), moderate negative correlation with ICTP (*ρ* =  − 0.38, *p* < 0.05), and weak correlation with the number of low-energy fractures (*ρ* =  − 0.11, *p* < 0.001). Weight was moderately correlated with BMD L1–L4 (*ρ* = 0.51, p < 0.001). In addition, weight was weakly correlated with the lowest hip *T*-score (*ρ* = 0.20, *p* < 0.05) and with the number of low-energy fractures (*ρ* =  − 0.07, *p* < 0.05; Tables 3 and 4).

**Table 3:** Effects of patient height on bone density, bone turnover, and fractures

Variable	Correlation to height	*p* value	*N*
BMD L1–L4	0.21	0.011	139
ICTP	− 0.38	0.031	33
Number of low-energy fractures	− 0.11	< 0.001	988

*BMD (L1-L4)* bone mineral density of lumbar vertebrae 1 through 4, *ICTP* cross-linked carboxy-terminal telopeptide of type I collagen

**Table 4:** Effects of patient weight on bone density and fractures

Variable	Correlation to weight	*p* value	*N*
BMD L1–L4	0.51	< 0.001	139
Lowest hip *T*-score^a^	0.20	0.039	102
Number of low-energy fractures	− 0.07	0.019	1005

^a^Lowest hip *T*-score: Some patients had *T*-scores measured in both hips. For the present study, we analyzed the lower *T*-score between the two hip measurements (i.e., the lowest hip *T*-score)

## Discussion

The main finding of this study was the identification of predicting factors for a second hip fracture. Risk factors for a second hip fracture were female sex, a fracture of the femur neck, high FRAX scores, and low *T*-scores for the femur neck. In addition, another predicting factor for a second hip fracture was low ambulatory capacity. This finding was consistent with common knowledge that the lack of rehabilitation and reduced ambulatory skills after a first hip fracture could increase the occurrence of a second hip fracture, and that activities aimed to develop body balance and muscle strength prevent fractures and falls. The mean age of our cohort was quite high, 79.3 years, similar to the mean ages reported in earlier studies [1, 3, 4, 37]. However, in the previous studies, specific risk factors for the second hip fracture were not identified in older patients [36–38]. Despite the facts that the FRAX-risk calculator does not capture the individual risk of falls or include the number of the previous fractures, it is useful for assessing a patient´s risk for a new fragile fracture. In particular, the FRAX is useful for predicting the risk of a new hip fracture, which was observed in our population of patients with fragile hip fractures. According to the FRAX scores for the risk of a new hip fracture, we found that the risk was significantly higher in the group of individuals with a second hip fracture (FRAX = 19.4) compared to those with the first hip fracture (FRAX = 10.2). In the future, continued use of the FRAX tool should be encouraged for making treatment decisions [6, 11].

The hip *T*-score was significantly lower among patients with only one hip fracture compared to those with a second hip fracture. This finding could be explained by the commencement of osteoporosis medication and vitamin D and calcium supplements after the first fracture. At the time of the first hip fracture, only 53 patients (4.7%) used osteoporosis medication. However, 28.8% of patients with first hip fractures had used anti-osteoporosis medications at some time during their lives. In contrast, among patients with a second hip fracture, 32% had previously used an anti-resorptive drug. Batin et al. (2018) showed that 8% of patients with hip fractures sustained a second hip fracture within a 5-year period [37]. In our study, a second hip fracture occurred within 1 year, in 2.9% of patients (*N* = 33). The positive effects of commencing anti-resorptive drugs and vitamin D supplements (like an increase in BMD) are typically detectable after 1–2 years [25, 39].

Low levels of vitamin D may lead to bone loss, increased bone resorption, fractures, and falls [21]. However, our study setting did not reveal the positive effects of vitamin D supplementation, due to the osteoporosis screening and treatment protocol for fragile hip fractures at Oulu University Hospital. For all patients with a first low-energy fracture, vitamin D supplementation was commenced, and the vitamin D level was titrated to ≥ 75 nmol/l, which was the level recommended for treating osteoporosis. Consequently, due to vitamin D supplementation after the first fracture, the *T*-scores were higher in patients with low vitamin D levels (i.e., the first hip fracture group) than in patients with high vitamin D levels (i.e., the second hip fracture group).

In this study, over half of the patients with fragile hip fractures had ionized calcium levels within the reference range. However, 40% had levels below the reference range. These findings were consistent with the previous studies which showed that low ionized calcium levels were generally associated with a higher risk of fractures [21]. However, the majority of our patients had vitamin D levels within the reference range (50–74 nmol/l). To interpret this result, it must be considered that the recommended vitamin D values for patients with osteoporosis are actually higher than the reference values (> 75 nmol/l), and only one-quarter (24%) of the patients in this study had vitamin D levels > 75 nmol/l at the time of the fracture [6]. Conversely, 76% of our patients had vitamin D levels lower than the recommended values for patients with osteoporosis. Consequently, our results were consistent with current knowledge that low levels of vitamin D are common among older people and could lead to fractures and falls [21].

Previous studies have shown that BMI had a protective effect on the risks of fracture and falling. This effect was explained by bone loading, which is sensed by osteocytes through the sclerostin pathway [35, 40]. In the present study, we could not detect a positive correlation between BMI and the hip *T*-score. This lack could be explained by a few strongly deviating values and missing data; DXA was measured in only about 10% of our patients.

Although bone turnover was previously associated with an increased risk of fractures [16, 17], we found that 37% of patients did not have a high bone turnover rates. This finding supported the hypothesis that not all patients with low-energy fractures have an elevated bone turnover rate. Thus, in contrast to conclusions drawn in the previous studies [26, 27], anti-resorptive medications might not benefit all patients with osteoporosis. We found a positive correlation between age and ICTP, consistent with the osteoporosis treatment protocol, which advises commencing anti-resorptive drug treatment in older patients with fragile fractures. This correlation between age and ICTP supported the notion that bone turnover markers could be used to assess the risk of fracture, consistent with findings from previous studies [2, 9, 12–15]. Moreover, we found that ALP levels were higher in patients that used ambulatory aids or lived in an institutional care facility. Taken together, these results suggested that bone turnover is likely to be high in older patients with hip fractures that live in institutional care facilities. Several previous studies have supported the clinical use of bone turnover markers in the treatment of osteoporosis; for example, they might be useful in evaluating fracture risk and patient commitment to treatment.

Surprisingly, we found that patient height was positively correlated with the BMD L1–L4, and it was moderately negatively correlated with ICTP and the number of fractures. These findings could be explained purely by axial loading on the lumbar vertebrae. Consistent with this finding, Kim et al. (2017) found a positive correlation between height and trabecular bone scores in a female cohort [41]. However, in overweight individuals, the force vectors of loading are not homogeneous. Although BMI was positively correlated with BMD, it has negative effect on bone quality. However, in studies that investigated changes in height and BMD, a loss of height was identified as an effective predictor of low BMD; consequently, changes in height might predict osteoporosis-related fractures [41–43].

Our study had some limitations. First, due to the retrospective nature of the study, substantial amounts of data were missing. For example, bone turnover markers were only measured in a small proportion of patients, which reflected the normal assessment protocol in the orthopedic ward. In our busy orthopedic unit, the so-called osteoporosis laboratory screening samples were not always collected before the hip surgery, as per instructions. Other laboratory values, like vitamin D, were not properly monitored in all patients, which restricted data collection. In addition, delays in laboratory tests might have induced strong deviations in a few values; for example, both PTH and PINP are very sensitive to circadian rhythms, immobilization, and the fracture healing status. In addition, not all patients underwent screening for osteoporosis, due to the lack of nurses that could carry out osteoporosis screening, and because patients in nursing homes could not undergo DXA measurements.

## Conclusion

We found several predictive factors for a second hip fracture, including female sex, a fracture in the femur neck, a high FRAX score, low *T*-scores for the femur neck, and low ambulatory capacity. Additionally, low ionized calcium values were detected in 42% of patients with fragile hip fractures. However, laboratory values were highly variable among patients with hip fractures. In addition, over one-third of patients did not exhibit high bone turnover. Thus, osteoporosis treatments should be planned carefully for individual patients. We found that only one-third of patients with a second hip fracture had been taking anti-osteoporosis treatments. These findings suggested that it is essential to establish systematic screening for osteoporosis in patients with a first fragile hip fracture.
